# Acculturation and self-rated health among Chinese and Korean immigrants aged 49 to 75

**DOI:** 10.3389/fpubh.2023.1272428

**Published:** 2023-12-19

**Authors:** Soomin Ryu, Brittany N. Morey, Yuxi Shi, Sunmin Lee

**Affiliations:** ^1^Department of Epidemiology, School of Public Health, University of Michigan, Ann Arbor, MI, United States; ^2^Program in Public Health, Department of Health, Society, and Behavior, University of California, Irvine, Irvine, CA, United States; ^3^Department of Medicine, School of Medicine, University of California, Irvine, Irvine, CA, United States

**Keywords:** acculturation, English proficiency, ethnic identity, self-rated health, Asian Americans, immigrants, Chinese Americans, Korean Americans

## Abstract

**Background:**

Given the rapidly growing Asian populations in the U.S. due to immigration, and the aging demographic of Asian immigrants, it is crucial to understand how acculturation shapes health among older adult Asian immigrants. We study the relationship between acculturation and self-rated health (SRH) and moderating roles of age and Asian subgroup.

**Methods:**

Our cross-sectional study consisted of 200 Chinese and 200 Korean immigrants aged 49–75 living in Baltimore–Washington DC metropolitan area, who were recruited from primary care physicians' clinics in Maryland and Northern Virginia. The participants completed the survey either in-person or by phone in their preferred language. Multidimensional proxies were used to measure acculturation: years living in the U.S. (≥23 years, <23 years), English proficiency (fluently/well, so-so, poorly/not at all), and ethnic identity (very Asian, mostly Asian, bicultural/westernized). SRH was measured using the question “How would you rate your general health?” (excellent/very good/good, fair/poor). Poisson regression models with robust error variance examined associations between acculturation and SRH, accounting for socioeconomic and health insurance factors.

**Results:**

Speaking English so-so and fluently/well had 0.73 (95% confidence intervals (CI): 0.55–0.97) and 0.51 (95% CI: 0.30–0.87) times the prevalence of having fair or poor SRH compared to speaking English poorly/not at all, respectively. The magnitudes and statistical significance of these associations were stronger among Chinese participants than Korean participants. Moreover, individuals who self-identified as bicultural/westernized had 0.63 times the prevalence of having fair or poor SRH (95% CI: 0.43–0.92) as those who self-identified as very Asian. The association was more pronounced among older participants (**≥**58) compared to younger participants (<58).

**Conclusion:**

Further research should identify the possible mechanisms linking acculturation with health to find effective strategies to enhance health among aging Asian immigrant populations.

## Introduction

Many previous studies on acculturation and health among immigrants have found support for a “healthy immigrant effect”—the paradox that recent immigrants have better health profiles than their native-born counterparts in the host country and that recent immigrants' health erodes with increasing time since migration (1–3). Acculturation is generally defined as “all changes that arise as a consequence of contact between individuals and groups of different cultural backgrounds (4)”. Overall, a higher level of acculturation among immigrant groups has been associated with a higher risk of unhealthy behavior such as drinking (5) as well as chronic diseases (6) including obesity (7–9), breast cancer (10), and diabetes (11–13). This may be because immigrants converge to the health profile of the host population due to the adoption of dietary patterns, health-related norms, and other health behaviors during the acculturation process (2, 14). Moreover, acculturation may have negative effects on health of immigrants because combining the ethnic culture with the mainstream culture is a stressful process (15, 16). However, acculturation can be also beneficial in terms of adaptability to multiple cultural environments and abilities, which benefit mental wellness (16). As acculturation is a complex and dynamic process and immigrants are diverse (17, 18), exact changes that occur during acculturation remain unclear (19, 20).

Examining the healthy immigrant effect among Asian Americans is important, as 6 out of 10 Asian Americans were born outside of the United States (U.S.) (21). Asians are the fastest-growing racial/ethnic group in the U.S.: Asians grew 81% between 2000 and 2019, from 10.5 million to 18.9 million, while the number is projected to increase to 35.8 million by 2060, more than triple Asians' population in 2000 (22). Considering the continuous growth of Asian Americans, it is crucial to study how acculturation shapes Asian immigrants' health over time.

The evidence for greater acculturation leading to worse overall health among Asian immigrant populations has used self-rated health (SRH) to compare more and less acculturated groups with regards to their health. SRH is increasingly used to evaluate immigrant health as it is a strong indicator of overall health outcomes linked to morbidity and mortality (23, 24). It has been a quick and useful measure that enables comparisons across datasets and studies, including studies among Asian immigrants (2). Prior research has supported the healthy immigrant effect of the negative association between acculturation and SRH. Longer duration of residence in the U.S. was associated with worse SRH among nationally representative samples of U.S.-born and foreign-born Asian Americans (2) as well as among samples of only foreign-born Asian immigrants in the U.S. (1, 25). Using a sample from the New Immigrant Survey that included Asian, Black, Hispanic, Multiracial, and White immigrants, Lee and colleagues also showed that longer duration in the U.S. was associated with reported worsening of SRH since moving to the U.S. (26).

On the contrary, other evidence has suggested that acculturation is associated with more optimal SRH. One study found that Chinese immigrants who lived in the U.S. longer than 5 years were more likely to report better SRH than those living in the U.S. for <5 years (27). Using a sample of U.S.-born and foreign-born Asian Americans and accounting for potential cohort confounding, Ro and colleagues demonstrated that a longer duration of stay in the U.S. was associated with lower odds of reporting poor-to-fair SRH (28). In the previously cited studies, length of residence was used as a proxy for acculturation. This assumes that the longer that Asian immigrants live in the U.S., the more acculturated they become. Prior studies may have been mixed in their findings because length of residence is an imperfect proxy for acculturation. Immigrants may not necessarily undergo acculturation simply due to living longer in the U.S. This begs the question of what it is about living in the U.S. as an immigrant that affects better or worse overall health.

Acculturation measured by language proficiency has more consistent findings. English proficiency and preference were associated with better SRH among samples of Asian immigrants (29, 30) and combined samples of U.S.-born and foreign-born Asian Americans (14). Another study also suggested that Chinese, Korean, Vietnamese, and Filipino Americans with low English proficiency were more likely to have fair or poor SRH compared to English proficient non-Hispanic White Americans (31). Kimbro and colleagues suggested that bilingual Asian immigrants were more likely to have physically and mentally better SRH compared to those who were Asian language dominant (32). A study used ethnic identity as another proxy for acculturation, reporting that U.S.-born and foreign-born Asian Americans who identified themselves as American were 3.5 times more likely to report good SRH than those who self-identified as Asian (14). To capture variations in acculturation experiences, two prior studies classified Asian immigrants into multiple categories based on several measures of acculturation, suggesting consistent findings that acculturation to the U.S. was associated with better SRH (33, 34).

Examining the role of acculturation on SRH may be especially important among older Asian immigrants, a growing population with health needs. Low English proficiency was found to be consistently associated with poor SRH among older adult Asian immigrants (29, 30) as well as combined samples of U.S.-born and foreign-born Asian Americans (35). For the length of residence in the U.S., Lam and colleagues suggested that the older adult Asian Americans who immigrated later in life had poorer self-rated physical health outcomes, whereas there was no significant association for mental health (36). Ro and colleagues also demonstrated that SRH improved with longer duration in the U.S. particularly among older cohorts of Asian immigrants (28). A recent study reported the moderating role of age in the association between the acculturation and SRH with different patterns by gender: the probability of having poor to fair SRH increased with age among less acculturated or bicultural Asian immigrant women, whereas the probability increased steeply with age among the highly acculturated Asian immigrant men (34). Older Asian immigrants are more likely to suffer from isolation and limited utilization of health services than their younger counterparts (30, 37, 38) which may result in poor SRH.

In addition to age, the country of origin may influence the relationship between acculturation and health. Westernization and its effects vary by country of origin because each country and ethnic group has different economic, political, and cultural backgrounds (32, 39, 40). These different histories influence exposure to Western culture, even prior to migration, making it likely that specific ethnic groups experience acculturation differently (41). Nevertheless, much less attention has been given to the heterogenous differences between Asian ethnic subgroups (32). A few studies highlighted differential associations of acculturation with health across Asian subgroups. For example, Park and colleagues showed that a longer duration of stay in the U.S. was significantly associated with increased alcohol use among people who immigrated from the Philippines, but not among people who immigrated from China or Vietnam, indicating the importance of Asian subgroup analysis (40). For SRH, Kimbro and colleagues found a stronger association between high language proficiency and better SRH among people who immigrated from the Philippines than people who immigrated from China or Vietnam (32).

Given the rapidly growing Asian populations in the U.S. particularly due to immigration, and the aging demographic of Asian immigrants, it is crucial to understand how acculturation shapes perceived health among older adult Asian immigrants (34). Relying on a single measure of acculturation (e.g., duration in the U.S.) may paint an incomplete picture. As acculturation is a dynamic, multifaceted, and complex construct (42), using several measurements should more effectively capture the level of acculturation (43, 44). The current study examined the association between acculturation and SRH among foreign-born older Chinese and Korean immigrants aged 49–75 and tested moderating roles of age and Asian subgroup using multidimensional proxies of acculturation: years living in the U.S., English proficiency, and ethnic identity. Considering that the positive relationship between acculturation and health has been relatively consistent among older adult Asian immigrants (28, 30, 35, 36), we hypothesized that more acculturated older adult Chinese and Korean immigrants who lived in the U.S. for longer, had high English proficiency and westernized ethnic identity would be less likely to report worse SRH. We also hypothesized that the magnitude and statistical significance of the associations between acculturation and SRH would be stronger among older Chinese and Korean immigrants than younger immigrants and there would be different associations by Asian subgroup.

## Materials and methods

### Sample

We used the baseline survey data from a randomized controlled trial to increase colorectal cancer (CRC) screening among 400 Chinese and Korean American immigrants (200 Chinese and 200 Korean immigrants). Study participants were recruited from primary care physicians' clinics in Maryland and Northern Virginia, and they were between the ages of 49 and 75, living in the Baltimore-Washington DC Metropolitan Area. All participants completed the survey between August 2018 and June 2020 either in-person or by phone in their preferred language (Mandarin, Korean, or English). Eighty-nine percent of the participants completed a self-administered questionnaire in-person, and 11 percent of the participants completed a research assistant-led phone survey due to the COVID-19 outbreak in March 2020. In-person survey participants signed informed consent forms during the meeting, while phone survey participants submitted consent forms prior to the survey via mail. Participants were able to get help from research assistants if they had any questions about survey items. This study was approved by the Institutional Review Boards of the University of Maryland, College Park and the University of California, Irvine.

### Self-rated health

Our dependent variable, SRH, was assessed using a question, “Would you say that in general your health is excellent, very good, good, fair or poor?” SRH is a widely used predictor of various health outcomes including morbidity (23) and mortality (24) and health behaviors such as smoking and alcohol consumption (45), and it has been applied in diverse populations including Chinese and Korean American immigrants (46). Responses were dichotomized into “excellent/very good/good” and “fair/poor” based on the previous studies (46, 47).

### Acculturation

We measured acculturation using three variables: years living in the U.S., English proficiency, and ethnic identity. Years living in the U.S. was calculated using the year difference between the date of baseline survey and the date of arriving in the U.S. We classified years living in the U.S. into two categories using 23 years (median) as a cutoff (13). As a sensitivity analysis, we repeated the same analysis using years in the US as a continuous variable and 3-categorical variable ( ≤ 10 years [11.8%], 11–20 years [28.5%], ≥21 years [59.8%]), with similar results (results not shown, but available upon request). English proficiency was evaluated using a question, “How well do you speak English?” Responses were recoded into three categories: “fluently like a native speaker/well,” “so-so,” and “poorly/not at all.” Ethnic identity was assessed using the question, “How would you rate yourself?” Available response options were “very Asian,” “mostly Asian,” “bicultural,” “mostly westernized,” or “very westernized” (48, 49). Few people rated themselves as mostly westernized (1.25% of the sample) or very westernized (0.25% of the sample), so we combined them with people identifying as bicultural. The ethnic identity variable used in analysis included three categories: very Asian, mostly Asian, or bicultural/westernized.

### Covariates

Age, gender, Asian subgroup, marital status, education, household income, employment status, and health insurance were included in the analysis. Age was used as a four categorical variable (<55, 55–59, 60–64, ≥65), gender was classified as male and female, and Asian subgroup was categorized as Chinese and Korean. Marital status was used as a binary variable: married/cohabiting and not currently married. Education was recoded into five categories: less than high school, high school graduate or GED, business/vocational school/some college, college graduate, and attended graduate/professional school. Household income was treated as six categories: <$20,000, $20,000–39,999, $40,000–59,999, $60,000–79,999, $80,000–99,999, and ≥$100,000. Employment status was categorized as full time, part time, and not employed. Health insurance status was recoded as private health insurance, Medicare/Medicaid, and no health insurance.

### Statistical analysis

First, descriptive analysis was conducted for the sample overall and stratified by SRH. Means and standard errors were reported for continuous variables and frequencies and percentages were reported for categorical variables. Two sample t-tests for continuous variables and chi-square tests for categorical variables were conducted to compare the differences by SRH. Second, Poisson regression models with robust error variance were used to estimate associations between acculturation variables and SRH. Poisson regression models were used in lieu of logistic models because logistic models may overestimate the associations using odds ratios when the outcome variable is prevalent at >10% (50, 51). We conducted a regression model for each acculturation variable, which included age, gender, Asian subgroup, and marital status, education, household income, employment status, and health insurance status. In the final model, we included all three acculturation variables together, adjusting for all covariates. Third, we tested age (using median age: 58) and Asian subgroup as potential effect modifiers for the associations between three acculturation variables and SRH. For interaction terms with p <0.1, we presented stratified analyses (non-significant findings with *p* ≥ 0.1 are not shown, but available upon request). All statistical analyses were computed using SAS version 9.4.

## Results

Table 1 presents the characteristics of the study participants. Of the 400 participants, 223 (55.8%) were classified as excellent/very good/good SRH, while 177 (44.2%) were classified as fair/poor SRH. There was a significant difference in English proficiency between two SRH groups: 32.7% of participants who had better SRH spoke English fluently like a native speaker/well, while only 10.2% participants who had worse SRH did. Also, participants with better SRH reported a significantly lower proportion of self-identifying as very Asian (51.6%) compared to those with worse SRH (71.8%). In addition, those that reported excellent/very good/good SRH had higher proportions of male (53.4%), attended graduate/professional school (34.5%), ≥$100,000 household income (36.8%), working full time (64.1%), and had private health insurance (66.4%) compared to those with fair/poor SRH (male: 39.6%, attended graduate/professional school: 11.3%, ≥$100,000 household income: 14.7%, working full time: 49.7%, private health insurance: 53.7%).

**Table 1:** Characteristics of the study participants (***n*** **=** 400).

		**Self-rated health**		
	**Total**	**Excellent/very good/good**	**Fair/poor**	***P*** **-value**
	***n*** = **400 (100%)**	***n*** = **223 (55.75%)**	***n*** = **177 (44.25%)**	
**Years living in the US**, ***n*** **(%)**				
<23 years	200 (50.0)	103 (46.2)	97 (54.8)	0.0870
≥23 years	200 (50.0)	120 (53.8)	80 (45.2)	
**English proficiency**, ***n*** **(%)**				
Fluently/well	91 (22.8)	73 (32.7)	18 (10.2)	<0.0001
So-so	148 (37.0)	89 (39.9)	59 (33.3)	
Poorly/not at all	161 (40.3)	61 (27.4)	100 (56.5)	
**Self-identity**, ***n*** **(%)**				
Very Asian	242 (60.5)	115 (51.6)	127 (71.8)	<0.0001
Mostly Asian	62 (15.5)	36 (16.1)	26 (14.7)	
Bicultural/mostly Westernized/very Westernized	96 (24.0)	72 (32.3)	24 (13.6)	
**Age**, ***n*** **(%)**				
<55	141 (35.3)	87 (39.0)	54 (30.5)	0.2370
55-59	86 (21.5)	47 (21.1)	39 (22.0)	
60-64	103 (25.8)	50 (22.4)	53 (29.9)	
≥65	70 (17.5)	39 (17.5)	31 (17.5)	
**Gender**, ***n*** **(%)**				
Female	211 (52.8)	104 (46.6)	107 (60.5)	0.0060
Male	189 (47.3)	119 (53.4)	70 (39.6)	
**Asian subgroup**, ***n*** **(%)**				
Chinese	200 (50.0)	119 (53.4)	81 (45.8)	0.1310
Korean	200 (50.0)	104 (46.6)	96 (54.2)	
**Marital status**, ***n*** **(%)**				
Not currently married	59 (14.8)	29 (13.0)	30 (17.0)	0.2691
Married/cohabit	341 (85.3)	194 (87.0)	147 (83.1)	
**Education**, ***n*** **(%)**				
Less than high school	43 (10.8)	20 (9.0)	23 (13.0)	<0.0001
High school graduate or GED	91 (22.8)	37 (16.6)	54 (30.5)	
Business/vocational school/some college	68 (17.0)	34 (15.3)	34 (19.2)	
College graduate	101 (25.3)	55 (24.7)	46 (26.0)	
Attended graduate/professional school	97 (24.3)	77 (34.5)	20 (11.3)	
**Household income**, ***n*** **(%)**				
<$20,000	62 (15.5)	22 (9.9)	40 (22.6)	<0.0001
$20,000–39,999	64 (16.0)	26 (11.7)	38 (21.5)	
$40,000–59,999	85 (21.3)	43 (19.3)	42 (23.7)	
$60,000–79,999	49 (12.3)	29 (13.0)	20 (11.3)	
$80,000–99,999	32 (8.0)	21 (9.4)	11 (6.2)	
≥$100,000	108 (27.0)	82 (36.8)	26 (14.7)	
**Employment**, ***n*** **(%)**				
Working full time	231 (57.8)	143 (64.1)	88 (49.7)	0.0146
Working part time	84 (21.0)	39 (17.5)	45 (25.4)	
Not currently working	85 (21.3)	41 (18.4)	44 (24.9)	
**Health insurance**, ***n*** **(%)**				
Private health insurance	243 (60.8)	148 (66.4)	95 (53.7)	0.0309
Medicare/Medicaid	74 (18.5)	37 (16.6)	37 (20.9)	
No health insurance	83 (20.8)	38 (17.0)	45 (25.4)	

SE, standard error.

Table 2 shows the results of Poisson regression models to estimate the associations between three acculturation variables and SRH. The adjusted prevalence ratios (aPR) and 95% confidence intervals (CI) are reported. In Model 1, duration of living in the U.S. was not associated with reporting fair or poor SRH (aPR: 0.94 95% CI: 0.74–1.18). In Model 2, individuals who spoke English so-so and fluently like a native speaker or well had 0.73 (95% CI: 0.56–0.95) and 0.48 (95% CI: 0.28–0.81) times the prevalence of having fair or poor SRH compared to those who spoke English poorly or not at all. In Model 3, individuals who self-identified as bicultural/westernized were less likely to have fair or poor SRH compared to those who identified as very Asian (aPR: 0.58, 95% CI: 0.39–0.85). When three measures of acculturation were included together in Model 4, English proficiency and ethnic identity were associated with SRH. Participants speaking English so-so and those speaking English fluently like a native speaker or well had 0.73 (95% CI: 0.55–0.97) and 0.51 (95% CI: 0.30–0.87) times the prevalence of having fair/poor SRH compared to those speaking English poorly or not at all. Additionally, those who identified as bicultural/westernized had 0.63 times the prevalence of having fair or poor SRH (95% CI: 0.43–0.92) as those who identified as very Asian. However, years living in the U.S. was not associated with prevalence of fair or poor SRH.

**Table 2:** Poisson regression analyses: years living in the US, English proficiency, self-identity, and self-rated health (*n* = 400).

	**Fair/poor self-rated health**			
	**PR (95% CI)**			
	**Model 1**	**Model 2**	**Model 3**	**Model 4**
**Years living in the US**				
<23 years	1.00			1.00
≥23 years	0.94 (0.74–1.18)			1.13 (0.89–1.44)
**English proficiency**				
Fluently/well		0.48 (0.28–0.81)^**^		0.51 (0.30–0.87)^*^
So–so		0.73 (0.56–0.95)^*^		0.73 (0.55–0.97)^*^
Poorly/not at all		1.00		1.00
**Self–identity**				
Very Asian			1.00	1.00
Mostly Asian			0.85 (0.61–1.18)	0.87 (0.62–1.21)
Bicultural/mostly Westernized/very Westernized			0.58 (0.39–0.85)^**^	0.63 (0.43–0.92)^*^
**Age**				
<55	1.00	1.00	1.00	1.00
55–59	1.12 (0.83–1.52)	1.09 (0.82–1.46)	1.14 (0.84–1.54)	1.10 (0.82–1.47)
60–64	1.05 (0.78–1.40)	0.96 (0.72–1.28)	1.07 (0.80–1.42)	0.98 (0.73–1.31)
≥65	0.89 (0.61–1.30)	0.83 (0.57–1.21)	0.88 (0.59–1.30)	0.81 (0.55–1.20)
**Gender**				
Male	1.00	1.00	1.00	1.00
Female	1.27 (1.01–1.60)^*^	1.25 (1.00–1.57)^*^	1.28 (1.02–1.60)^*^	1.26 (1.01–1.58)^*^
**Asian subgroup**				
Korean	1.00	1.00	1.00	1.00
Chinese	0.93 (0.73–1.20)	0.91 (0.71–1.16)	1.10 (0.85–1.42)	1.06 (0.82–1.38)
**Marital status**				
Married/cohabit	1.00	1.00	1.00	1.00
Not currently married	1.06 (0.81–1.40)	1.04 (0.79–1.37)	1.14 (0.87–1.50)	1.10 (0.83–1.45)
**Education**				
Less than high school	1.82 (1.06–3.12)^*^	1.27 (0.72–2.24)	1.63 (0.95–2.82)^†^	1.18 (0.65–2.13)
High school graduate or GED	1.98 (1.22–3.23)^**^	1.42 (0.85–2.38)	1.83 (1.12–2.98)^*^	1.36 (0.80–2.30)
Business/vocational school/some college	1.87 (1.13–3.11)^*^	1.54 (0.92–2.57)	1.81 (1.10–3.00)^*^	1.51 (0.90–2.54)
College graduate	1.79 (1.10–2.92)^*^	1.45 (0.87–2.40)	1.70 (1.04–2.77)^*^	1.42 (0.85–2.37)
Attended graduate/professional school	1.00	1.00	1.00	1.00
**Income**				
<$20,000	1.86 (1.15–3.00)^*^	1.57 (0.97–2.55)^†^	1.70 (1.06–2.73)^*^	1.52 (0.94–2.45)^†^
$20,000–39,999	1.62 (1.01–2.59)^*^	1.36 (0.85–2.17)	1.56 (0.98–2.49)^†^	1.37 (0.86–2.19)
$40,000–59,999	1.37 (0.88–2.14)	1.17 (0.75–1.85)	1.38 (0.89–2.13)	1.21 (0.77–1.89)
$60,000–79,999	1.15 (0.68–1.93)	1.01 (0.60–1.72)	1.13 (0.67–1.90)	1.03 (0.61–1.73)
$80,000–99,999	0.97 (0.54–1.72)	0.86 (0.47–1.58)	0.96 (0.54–1.72)	0.89 (0.48–1.63)
≥$100,000	1.00	1.00	1.00	1.00
**Employment status**				
Working full time	1.00	1.00	1.00	1.00
Working part time	1.06 (0.82–1.38)	1.06 (0.82–1.37)	1.04 (0.80–1.35)	1.05 (0.81–1.35)
Not currently working	1.02 (0.76–1.37)	0.94 (0.70–1.27)	1.00 (0.74–1.34)	0.94 (0.70–1.27)
**Health insurance**				
Private health insurance	1.00	1.00	1.00	1.00
Medicare/Medicaid	1.00 (0.72–1.38)	1.04 (0.76–1.44)	1.05 (0.76–1.47)	1.08 (0.77–1.51)
No health insurance	1.15 (0.89–1.49)	1.14 (0.88–1.47)	1.15 (0.89–1.49)	1.14 (0.89–1.47)

^^†^^p <0.1, ^*^p <0.05, ^**^p <0.01.

R, prevalence ratio; CI, confidence interval.

We tested age and Asian subgroup as potential effect modifiers for the associations between three measures of acculturation and SRH using models adjusted for all sociodemographic variables. The stratified results are presented in Table 3 only if the effect modification results were significant at p <0.1. Age modified the association between ethnic identity and SRH. Therefore, we present stratified analysis by whether participants were <58 years-old or equal to or >58 years-old (the median age). Among the participants aged 58 or older, people who identified as bicultural/westernized were much less likely to have fair or poor SRH compared to those who were very Asian (aPR: 0.29, 95% CI: 0.12–0.67), while the association between ethnic identity and SRH among the participants aged younger than 58 was lesser in magnitude. We additionally conducted effect-modification analysis using a binary age group based on retirement age (65 years) and more narrow age groups (<55, 55–59, 60–64, ≥65). Although these age groups were not statistically significant effect-modifiers, the stratified analysis confirmed that the magnitude of negative associations between being bicultural/westernized and fair/poor SRH were pronounced among participants who were older compared to those who were younger (Appendix 1). Asian subgroup modified the association between English proficiency and SRH. Among the Chinese participants, those who spoke English fluently like a native speaker/well or so-so were less likely to have fair or poor SRH (fluently like a native speaker/well: aPR: 0.33, 95% CI: 0.14–0.78; so-so: aPR: 0.46, 95% CI: 0.29–0.73) compared with those who speak English poorly/not at all. These associations were not statistically significant among the Korean participants, but still in the same direction.

**Table 3:** Stratified analysis: English proficiency, self-identity, and self-rated health (*n* = 400).

	**Fair/poor self-rated health**	
	**PR (95% CI)**	
	**Korean** ^a^	**Chinese** ^a^
	***n*** = **200**	***n*** = **200**
**English proficiency**		
Fluent like a native speaker/well	0.63 (0.33-1.21)	0.33 (0.14-0.78)^*^
So-so	0.90 (0.63-1.27)	0.46 (0.29-0.73)^***^
Poorly/not at all	1.00	1.00
	**Age** **<** **58**^**b**^	**Age** **≥** **58**^**b**^
	***n*** **=** **197**	***n*** **=** **203**
**Self-identity**		
Very Asian	1.00	1.00
Mostly Asian	0.85 (0.50–1.45)	0.92 (0.60–1.43)
Bicultural/mostly Westernized/very Westernized	0.78 (0.49–1.24)	0.29 (0.12–0.67)^**^

^a^English proficiency + age, gender, marital status, education, household income, employment status, health insurance.

^b^Self-identity + gender, Asian subgroup, marital status, education, household income, employment status, health insurance.

PR, prevalence ratio; CI, confidence interval.

^*^p <0.05, ^**^p <0.01, ^***^p <0.001.

## Discussion

With the rapid growth and eventual aging of Asian immigrants, it is essential to study the health and overall wellbeing of older Asian immigrants as they adapt to the U.S. (52). This paper provides findings about the association between multidimensional acculturation and SRH among older Chinese and Korean American immigrants and differential associations by age and Asian subgroup.

Our findings suggest that higher acculturation measured by English proficiency and ethnic identity was associated with better SRH among older adult Asian immigrants. Individuals with higher English proficiency and who identified as bicultural or westernized were less likely to report poor to fair SRH compared to their less acculturated counterparts. These results are consistent with growing evidence that greater acculturation is associated with better SRH among Asian immigrants. Previous studies suggested that English proficiency was consistently associated with better SRH among samples of Asian immigrants (29, 30, 32) and in combined samples of U.S.-born and foreign-born Asian Americans (14, 31). For ethnic identity, a prior study also found that U.S.- and foreign-born Asians who self-identified as American were 3.5 times more likely to have good SRH than those who self-identified as Asian (14). By contrast, our result suggested that the association between the length of residence in the U.S. and odds of poor SRH was not statistically significant after adjusting for socioeconomic status covariates or including other acculturation proxies. Our finding is consistent with a previous study that reported insignificant association between duration of time in the U.S. and SRH among Asian immigrants (32). However, the finding is contrary to some studies that showed a positive association of duration of residence with poor SRH among Asian Americans (1, 2), which included both U.S.-born and foreign-born Asian Americans. Together, our findings suggest that acculturation is a complex construct that should be assessed using multidimensional measures.

Our results are consistent with previous studies on acculturation and SRH, while they contrast with the traditional knowledge about the erosion of immigrant health since migration, which suggests that acculturation is associated with unhealthy behaviors and chronic diseases (1–3, 5–8, 13) as immigrants adopt dietary patterns, health-related norms and behaviors in the U.S. (2, 14). The result for SRH is different probably because SRH is a holistic and subjective indicator of perceived physical, mental, emotional health, which depends on external factors and internal responses (32, 53). For example, acculturative stress is prominent for less acculturated Asian Americans, which contributes to poor mental health and ultimately worse SRH despite their lower prevalence of major diseases compared to more acculturated populations (14). Additionally, more acculturated Asian Americans are more likely to receive health screening and healthcare services than less acculturated Asian Americans (14, 32, 34, 54). In particular, higher English proficiency and more bicultural or western identity—the acculturation factors most associated with better SRH—are likely associated with higher health literacy and better access to health care, as structural barriers to access will be easier for those individuals to overcome (55–57).

Additionally, we also found that age and Asian subgroup were effect modifiers for the associations between acculturation and SRH using models adjusted for all covariates. The negative association between English proficiency and poor to fair SRH was more pronounced among Chinese immigrants than Korean immigrants. To our knowledge, this is the first study to demonstrate differences between Chinese and Korean Americans in this association. It may be that English proficiency confers better health for Chinese immigrants more so than for Korean immigrants due to a greater ability to access health promoting resources in English in the study area for Chinese compared to Korean populations. This finding is consistent with previous studies that found the heterogeneity of Asian immigrants in the associations between acculturation and health behaviors or outcomes (32, 39, 40). The process and effects of acculturation are diverse across the country of origin due to different reasons and contexts for migration, economic and political circumstances of host countries, ethnic differences in their social networks, as well as cultural differences in the conception of health status (32, 39, 40).

Age modified the association between ethnic identity and SRH, such that there was a stronger health protective effect of identifying as bicultural/westernized vs. very Asian for people at older ages than those at younger ones. This finding is consistent with several previous studies that reported a positive association between acculturation and SRH among older adult Asian immigrants (28, 36). It is noteworthy that the sample was generally older to begin with, with ages ranging from 49 to 75, but we still found the difference by age group. It is likely that as age increases, older Asian immigrants are more likely to suffer from cultural and social isolation and limited access to culturally appropriate health services (30, 37, 38). Therefore, for much older Asian immigrants, identifying as bicultural or westernized may have a more pronounced protective effect on health, since their social networks are broader or it is easier for them to access the health and social services they need (46, 58).

This study has some limitations to highlight. First, we were not able to examine causal mechanisms between acculturation and SRH as the data was cross-sectional. Second, our study was limited to the Chinese and Korean immigrants aged 49–75 living in the Baltimore-Washington D.C. Area, who were not randomly selected. In particular, as we used survey data of CRC screening study, the exclusion and inclusion criteria related to CRC might have affected our study's results. For example, Chinese and Korean immigrants who had CRC diagnosis were excluded from our study. It is possible that we might have excluded participants who might have had lower SRH, which could have underestimated the true association. Therefore, the sample is not representative of the population of Chinese and Korean immigrants. Third, we did not have information about participants' health-promoting behaviors, such as receiving health screening or utilizing healthcare services, which would allow us to test their mediating role in the association between acculturation and SRH (14, 32, 34).

Nonetheless, the current study has public health implications for Asian American communities, policy makers, and medical professionals. By using multidimensional proxies of acculturation, the findings suggest that being less acculturated was associated with worse SRH overall among older adult Asian immigrants. Our study also highlights that the association was more pronounced among Chinese immigrants and older participants compared to Korean immigrants and younger participants. There is a critical need to develop intervention programs to improve health among less acculturated Asian immigrants, particularly older and Korean immigrants. For example, local governments and community centers may support in-language and culturally appropriate education programs, free or low-cost health services, and vital social supports. Classes to learn basic English might also be useful for interested Chinese and Korean immigrants. We also learned that duration of stay in the U.S. alone will not provide sufficient information for degree of acculturation. This is important given that many national-level surveys use duration of stay in the U.S. as a marker for acculturation.

Using a prospective study design, future research on this topic should investigate the causal relationship between acculturation and SRH and focus on finding potential mediators between the association between acculturation and SRH. It will provide levers for future intervention. Moreover, future studies need to examine differential associations across various Asian subgroups and a wide range of age groups as these findings will help to target future interventions. Furthermore, studies should carefully assess acculturation with various measurements (e.g., social interactions, attitudes, values). By doing so, we will find effective strategies to enhance health among aging Asian immigrant populations in the U.S.

## Data availability statement

The original contributions presented in the study are included in the article/supplementary material, further inquiries can be directed to the corresponding authors.

## Ethics statement

The studies involving humans were approved by Institutional Review Boards of the University of Maryland, College Park and the University of California, Irvine. The studies were conducted in accordance with the local legislation and institutional requirements. The participants provided their written informed consent to participate in this study.

## Author contributions

SR: Data curation, Formal analysis, Writing – original draft. BM: Formal analysis, Writing – review & editing. YS: Data curation, Formal analysis, Writing – review & editing. SL: Funding acquisition, Project administration, Supervision, Writing – review & editing.

## Appendix

**Table:** Stratified analysis: self-identity and self-rated health by different age group (*n* = 400).

	**Fair/poor self-rated health**			
	**PR (95% CI)**			
	**Age <65**	**Age** **≥** **65**		
	***n*** **=** **330**	***n*** **=** **70**		
**Self-identity**				
Very Asian	1.00	1.00		
Mostly Asian	0.83 (0.57–1.21)	0.62 (0.26–1.45)		
Bicultural/mostly Westernized/very Westernized	0.66 (0.44–0.98)^*^	0.16 (0.03–0.87)^*^		
	**Age** **<** **55**	**55** **≤** **Age** **<** **60**	**60** **≤** **Age <65**	**Age** **≥** **65**
	***n*** **=** **141**	***n*** **=** **86**	***n*** **=** **103**	***n*** **=** **70**
**Self-identity**				
Very Asian	1.00	1.00	1.00	1.00
Mostly Asian	0.69 (0.34–1.39)	1.05 (0.44–2.50)	1.09 (0.62–1.92)	0.62 (0.26–1.45)
Bicultural/mostly Westernized/very Westernized	0.72 (0.40–1.30)	0.73 (0.35–1.54)	0.51 (0.20–1.34)	0.16 (0.03–0.87)^*^

Model: Self-identity + gender, Asian subgroup, marital status, education, household income, employment status, health insurance.

R, prevalence ratio; CI, confidence interval.

^*^p <0.05.
